# Complete genome sequence of two skunk adenovirus strains identified in African pygmy hedgehogs in Germany

**DOI:** 10.1128/mra.00328-26

**Published:** 2026-07-23

**Authors:** Hannah Leah Elbo Morito, Sonja T. Jesse, Sandra Runft, Christina Puff, Wolfgang Baumgärtner, Albert D. M. E. Osterhaus, Martin Ludlow

**Affiliations:** 1 Research Center for Emerging Infections and Zoonoses, University of Veterinary Medicine Foundation, Hannover, Germany; 2 Department of Pathology, University of Veterinary Medicine Foundation, Hannover, Germany; Portland State University, Portland, Oregon, USA

**Keywords:** adenovirus, mastadenovirus, skunk adenovirus type 1, African pygmy hedgehog, cross-species infection

## Abstract

We report complete genome sequences of two skunk adenovirus 1 (SkAdV-1) strains from African pygmy hedgehogs (*Atelerix albiventris*) in Germany. The genome sequences of 31,786 bp and 31,788 bp contain five indel sites previously noted in an SkAdV-1 strain from this host species but not from other species.

## ANNOUNCEMENT

Adenoviruses are ubiquitous DNA viruses (1, 2) that display a largely species-specific host tropism, although some mastadenoviruses, such as canine adenovirus type 1, can cross species barriers (3–6). Similarly, clinical disease caused by skunk adenovirus 1 (SkAdV-1) infection (7), has been reported in the gray fox (8), pygmy marmoset (9), raccoon (10), porcupine (10, 11), and African pygmy hedgehog in Japan and USA (12–15). Here, we report fatal cases from January 2019 of skunk adenovirus 1 (Species, *Mastadenovirus trianonense*) in African pygmy hedgehogs with catarrhal purulent bronchopneumonia in Lower Saxony, Germany.

DNA was isolated from frozen lung tissues using DNeasy Blood and Tissue Kit (QIAGEN, Hilden, Germany) and used as a template in a pan-adenovirus PCR, as previously described (16). Amplicons were purified using a QIAquick PCR & Gel Cleanup Kit (QIAGEN, Hilden, Germany) and Sanger sequencing (Microsynth Seqlab GmbH, Göttingen, Germany). NCBI BLASTn analysis (17) showed 98% nucleotide identity to skunk adenovirus PB1 strain (NC_027708). Libraries from two samples (S17/19 and S18/19) were prepared with the Nextera XT DNA Library Preparation Kit (Illumina, San Diego, CA, USA) and sequenced in Illumina NextSeq 550 with NextSeq 500/550 Mid Output Kit v2.5 Kit (300 cycles) at 2 × 75 bp paired-end reads, generating 10,142,420 reads (animal S17/19) and 14,597,926 reads (animal S18/19) with approximately 40× depth. Fastq files were imported to CLC Genomics Workbench 21.0.3 (CLC bio, Aarhus, Denmark), and reads were trimmed using default parameters. Reads were mapped using the default mapper algorithm in Geneious Prime 2024.0.4 (Biomatters Ltd., Auckland, New Zealand) with SkAdV-1 strain PB1 (GenBank accession no. NC_027708) as reference sequence, resulting in 34,931 and 105,120 mapped reads for S17/19 and S18/19, respectively. Variant nucleotide positions were identified using the built-in variant finder algorithm in Geneious Prime (Table 1).

**TABLE 1:** Nucleotide and amino acid variation in SkAdV-1 strains from African pygmy hedgehogs

		SkAdV-1 PB1 strain (NC_027708)		SkAdV-1 HO-2018 strain (MK937781)	
Position	Putative CDS	nt/aa change (S17/19)	nt/aa change (S18/19)	nt/aa change (S17/19)	nt/aa change (S18/19)
163	–^*a*^	G > C	–	G > C	–
1291	–	C > G	C > G	C > G	C > G
1809	E1B 19K	C > A/Q133K	C > A/Q133K	–	–
1809	E1B 55K	C > A/E19A	C > A/E19A	–	–
1831	E1B 19K,55K	C > T	C > T	–	–
2081	E1B 55K	T > A/Y103N	T > A/Y103N	–	–
2347	E1B 55K	T > C	T > C	–	–
5609	pol	G > T	G > T	–	–
6017	pol	C > T	C > T	–	–
6161	pol	A > G	A > G	–	–
6398	pol	C > T	C > T	–	–
8002	pTP	C > T	C > T	–	–
8857	pTP	A > G	A > G	–	–
10,894	52K	C > T/P374S	C > T/P374S	–	–
10,894	pIIIa	C > T	C > T	–	–
11,293	pIIIa	C > T	–	C > T	–
14,201	pVII	C > T	C > T	–	–
15,117	pV	T > A	T > A	–	–
15,803	pV	G > A/R395Q	G > A/R395Q	–	–
17,299	hexon	G > A	G > A	–	–
21,988	100k	–	–	insTCAGAC	insTCAGAC
21,999	100K	delCCACTACCT	delCCACTACCT	–	–
22,581 (22572)	100K	C > T	C > T	–	–
23,102 (23093)	100K	T > C/V409A	T > C/V409A	–	–
25,879 (25870)	E3 ORFa	delATGACATGC CAGACCCCTCC T C TTTCCATGATCTCTTTCACA	delATGACATGC CAGACCCCTCCTC TT TCCATGATCTCTTTCACA	–	–
27,394 (27343)	fiber	G > A	G > A	–	–
27,613 (27614)	fiber	delGGTGGG	delGGTGGG	–	–
28,440 (28383)	fiber	G > A/G507E	G > A/G507E	–	–
28,703 (28646)	fiber	G > A/D595N	G > A/D595N	–	–
28,731 (28674)	–	insC	insC	–	–
30,326 (30269)	E4 ORFc	G > A	G > A	–	–
30,517 (30460)	E4 ORFc	T > C	T > C	T > C	T > C
30,707 (30650)	E4 ORFb	G > A	G > A	–	–
30,976 (30919)	–	delAAAAAG	delAAAA	–	–
30,981 (30920)	–	–	G > A	–	–
31,715 (31653)	–	–	–	A > C	A > C

^*a*^ –, no change or not applicable.

The sequence identity of genome termini sequences was confirmed using a single primer (5′-CATCATCAATAATATACAGGAC-3′) based on the conserved sequence of mastadenovirus inverted terminal repeats, as a forward and reverse primer for PCR amplication of the 5′- and 3′- genome ends respectively. This primer was used in combination with a reverse primer (5′-CAGAGCTTTCAAGTAACAC-3′) or a forward primer (5′-CATAGAAGAATCGGGAAGAG-3′) for PCR amplification using NEB Q5 High Fidelity DNA Polymerase (New England Biolabs, Massachusetts, USA) of the 5′- or 3′ genome terminus, respectively. Resulting amplicons were gel purified and Sanger sequenced, as described above. This resulted in whole genome sequences of 31,786 bp (SkAdV-1/DE/S17/19) and 31,788 bp (SkAdV-1/DE/S18/19), with both genomes having 49% GC content. Putative coding regions were predicted using Artemis 18.2.0 (18) and annotated using Geneious Prime.

**Fig 1:**
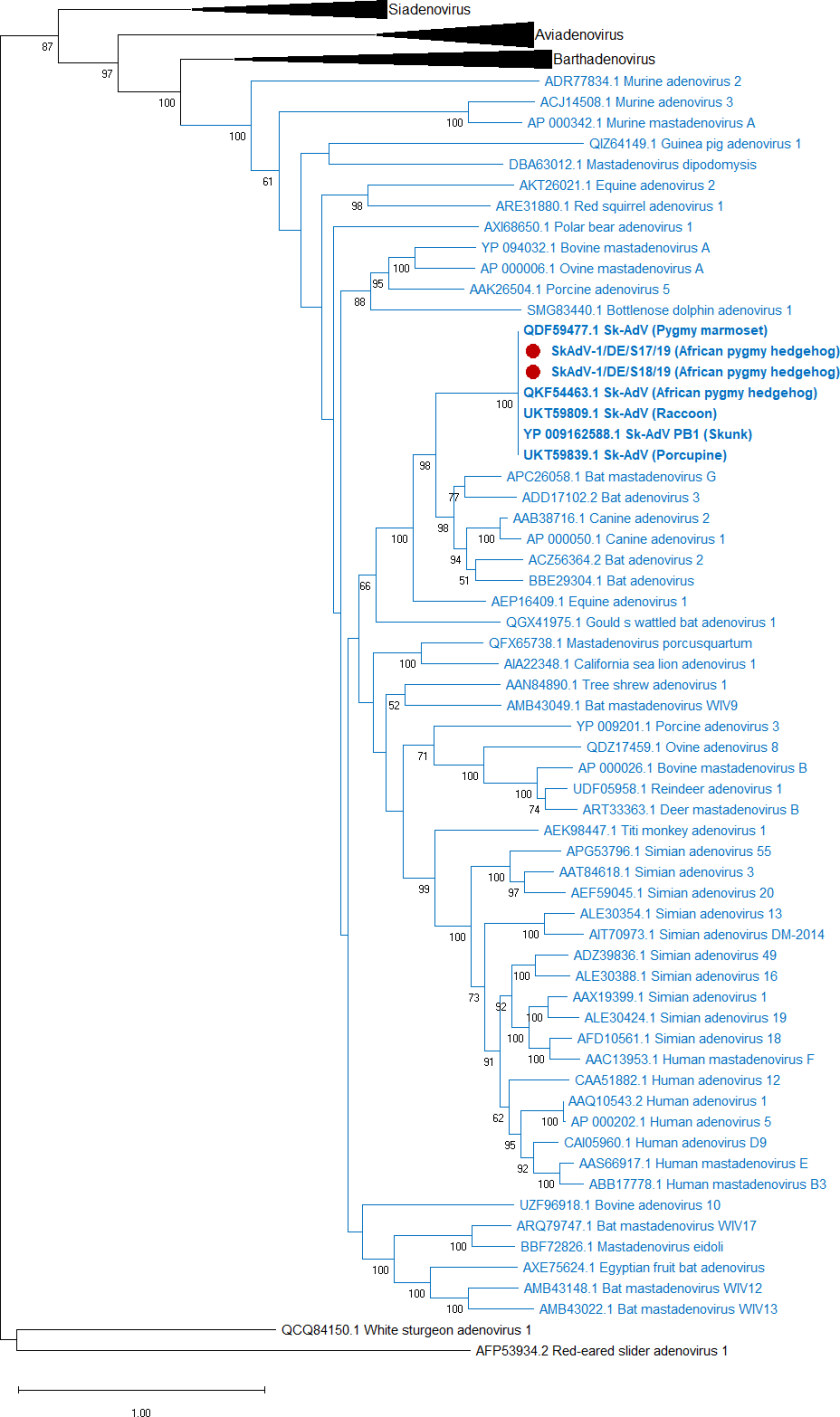
Phylogenetic analysis of the DNA polymerase amino acid sequence of representative members of each genus of the family *Adenoviridae*. Sequence alignment was performed using MAFFT7, with a maximum likelihood tree constructed using RAxML in DNASTAR MegAlign Pro 17.4.0.81. Branch support was assessed using 1,000 bootstrap replicates with values higher than 70% displayed using MEGA 11.0.13. SkAdV genome sequences (S17/19, S18/19) are indicated by red circles.

Pairwise nucelotide identity between the two genomes was 99.98% and were 99.8% identical to SkAdV-1 PB1 strain. Both genomes also had four deletion sites and one single nucleotide insertion site compared with the PB1 strain with these deletions shared with the HO-2018 strain from a Japanese African Pygmy hedgehog (13) (Table 1). In contrast, a six-nucleotide sequence at position 21,988 is shared with PB1 that is not present in HO-2018. Phylogenetic analysis of the DNA polymerase amino acid confirmed that SkAdV-1/DE/S17/19 and SkAdV-1/DE/S18/19 form a distinct cluster with SkAdV-1 strains identified in other mammals (Fig. 1). The observed close relationship to bat and canine adenoviruses is consistent with the suggestion of Kozak et al. (7) that SkAdV-1 shares a common ancestor with bat and canine adenoviruses.

## Data Availability

Consensus SkAdV-1/DE sequences have been deposited in GenBank under the accession numbers PZ095820 and PZ095821. The raw sequence data were deposited in the NCBI Sequence Read Archive under BioProject accession number PRJNA1433230 and SRA run accession numbers SRR37499769 and SRR37499770.
